# Applicability of Physiological Monitoring Systems within Occupational Groups: A Systematic Review

**DOI:** 10.3390/s21217249

**Published:** 2021-10-30

**Authors:** Denisse Bustos, Joana C. Guedes, João Santos Baptista, Mário P. Vaz, José Torres Costa, Ricardo J. Fernandes

**Affiliations:** 1 Associated Laboratory for Energy, Transports and Aeronautics, LAETA (PROA), Faculty of Engineering, University of Porto, 4200-465 Porto, Portugal; ldbs@fe.up.pt (D.B.); jccg@fe.up.pt (J.C.G.); gmavaz@fe.up.pt (M.P.V.); 2 Porto Biomechanics Laboratory, Faculty of Sport, University of Porto, 4200-450 Porto, Portugal; ricfer@fade.up.pt; 3 Associated Laboratory for Energy, Transports and Aeronautics, LAETA (PROA), Faculty of Medicine, University of Porto, 4200-319 Porto, Portugal; zecatoco@sapo.pt; 4 Center of Research, Education, Innovation and Intervention in Sport, Faculty of Sport, University of Porto, 4200-450 Porto, Portugal

**Keywords:** wearable sensors, occupational activities, occupational physiology, performance, cardiac reactivity, physical activity patterns, heat stress, physical exertion, fatigue

## Abstract

The emergence of physiological monitoring technologies has produced exceptional opportunities for real-time collection and analysis of workers’ physiological information. To benefit from these safety and health prognostic opportunities, research efforts have explored the applicability of these devices to control workers’ wellbeing levels during occupational activities. A systematic review is proposed to summarise up-to-date progress in applying physiological monitoring systems for occupational groups. Adhering with the PRISMA Statement, five databases were searched from 2014 to 2021, and 12 keywords were combined, concluding with the selection of 38 articles. Sources of risk of bias were assessed regarding randomisation procedures, selective outcome reporting and generalisability of results. Assessment procedures involving non-invasive methods applied with health and safety-related goals were filtered. Working-age participants from homogeneous occupational groups were selected, with these groups primarily including firefighters and construction workers. Research objectives were mainly directed to assess heat stress and physiological workload demands. Heart rate related variables, thermal responses and motion tracking through accelerometry were the most common approaches. Overall, wearable sensors proved to be valid tools for assessing physiological status in working environments. Future research should focus on conducting sensor fusion assessments, engaging wearables in real-time evaluation methods and giving continuous feedback to workers and practitioners.

## 1. Introduction

Miners and steelworkers are regularly exposed to high heat conditions [1,2]. Police officers and other first responders are called with little to no notice into situations of extreme danger and physiological stress [3]. Firefighters and soldiers often wear personal protective equipment that imposes additional thermal burdens from insulation and additional carried weight [4,5,6] and are exposed to high physical, mental and emotional stress levels for prolonged periods [7]. In general, these high-risk professions are repeatedly subjected to operations in austere environments and dysregulated sleep [3]. On the other hand, limited by the natural environment factors (e.g., construction workers [8]) or indoors cold exposure conditions (e.g., food industry workers [9]), some professions develop strenuous work while regularly exposed to thermal stress. In any of these cases, the characteristics of occupational activities demand an additional physiological effort involving thermoregulatory adaptation and physical endurance to cope with the load of their activities.

For example, heat stress is sometimes combined with physical activity, such as during military or firefighting simulation training and in theatre operations. In that case, the resulting struggle between muscle and skin (due to energetic demands and cooling thermoregulation, respectively) for a relatively limited cardiac output can become a significant fatigue contributor and exercise inhibitor relative to a cooler or within limits condition [10]. Chronic exposure to some of these occupational factors threatens the body efforts to maintain homeostasis and a balanced state. Physiological and psychological responses may begin to deteriorate, causing long-term adverse effects [11]. Literature has reported that many health related risks (e.g., heat-related illness and cardiovascular disease) may be higher than those in the general population [12], meaning that suitable prevention measures (e.g., recovery periods and acclimation time) are essential to prevent it.

As a result, occupational physiology related research has become fundamental to understand the physiological functions of the human body and its ability to cope with the work associated stresses. By investigating and assessing work relevant procedures, organisations can derive suggestions for modifying these processes focusing on health complaints prevention [13]. Previously, work–rest cycles and training periods could only be addressed using generalised models based on estimated inputs about individuals in standard conditions. The advent of wearable physiological and health monitoring devices might help overcome these limitations but needs the right questions in sourcing, developing, validating and applying such technologies [14].

Wearable physiological monitoring systems can help evaluate individuals’ health and performance from their real-time collection and analysis of physiological states [15,16]. This approach presents key improvements in population-based predictions derived from ambient conditions and the general context of a working activity. Advances in computing power and microelectronics enable these improvements in human performance assessment, with real-time physiological measurement capabilities and data processing to provide valuable information about the individual [17]. Currently available commercial systems include heart rate monitors [18], temperature sensors [19], accelerometers [20] and integrated sensors [21]. However, these apparatuses usually do not satisfy the requirements for more profound occupational research. When they offer something more than raw physiological data, computed information is generally based on proprietary algorithms that cannot be adequately validated, making the output unusable [17].

Thus, the critical component of a real-time physiological monitoring system is the process that transforms data into valuable knowledge for a worker or a small unit leader to manage its operational elements. Research and reviews on the application of these systems have increased [22], particularly in the sports and medical monitoring fields [23,24], and some within specific professionals (e.g., construction workers [25,26]). However, no comprehensive literature review gathering in-field physiological monitoring from a general occupational perspective was identified; particularly, a comprehensive search on variables selection and processing methods derived from regular working activities monitoring has not been conducted. Investigations in real stressful scenarios are fundamental to understand the effects of some working environments and have a clear perspective on the applicability of available sensors since they differ significantly from controlled locations regarding environment, activity and subject motivation.

To improve the quality and generalisability of research and overcome the limitations associated with in-field measurements, it is essential to structure what has been done within real-life occupational activities, among which occupational groups and with what degree of success. Hence, the current work proposes to systematically review the literature to: (i) summarise the physiological metrics that have the potential to be measured in real-time among occupational groups; (ii) determine the general research objectives in which these physiological variables have been measured and the occupational groups covered; (iii) compile used physiological sensors and the corresponding extracted information; and (iv) discuss current trends and potential challenges of these systems applications, identifying the research gaps that can be addressed in future works.

## 2. Methods

This systematic review was conducted following the Preferred Reporting Items for Systematic reviews and Meta-Analyses (PRISMA) Statement [27]. Based on related methodology [28,29], a protocol [30] (registered in PROSPERO under CRD42019119787) was prospectively elaborated to determine adequate guidelines for retrieving relevant results.

The search was developed firstly through electronic databases: Scopus, Science Direct, Academic Search Ultimate, Web of Science and PubMed. The first four are among the largest multidisciplinary abstract and citation databases of peer-reviewed literature. Furthermore, since the goals and selected keywords are associated with health-related conditions within occupational groups, PubMed (the most complete resource for literature in medicine and biological sciences) was also consulted. The search was performed using relevant search terms formed by two groups of keywords related to monitoring procedures and their potential applicability: (i) “physiological monitoring”, “noninvasive monitoring”, “medical monitoring” and “wearable sensors” and (ii) “assessment”, “occupational”, “model”, “fatigue”, “algorithm”, “worker”, “training” and “physical exertion”.

Keywords from both groups were combined as follows: (((“physiolog*monitor*”) OR (“noninvasive monitor*”) OR (“medical monitor*”) OR (“wearable sens*”)) AND ((assessment) OR (occupational) OR (model) OR (fatigue) OR (algorithm) OR (worker) OR (training) OR (“physical exertion”))). The query was tested and adapted for each database as presented in the protocol [30]. It was applied in the title, abstract and keywords for Scopus and Science Direct. Title and Abstract were the search fields for PubMed and Academic Search Ultimate, and Topic was selected in Web of Science. Later, the search continued by checking reference lists of initially retrieved papers and applying the snowballing technique [31] until no more relevant information was found. Finally, additional sources found in citations were consulted.

The study selection was also based on three phases of exclusion. Initially, filters from databases were applied, and studies were limited by date (2014–2021), document type (research articles), source type (peer-reviewed journals) and language (English written papers). The main goal of this stage was to gather the most relevant up-to-date information. However, when looking through the first collected records’ references, the initially delimited timeframe was no longer considered. Then, as a second exclusion phase, repeated records were eliminated. Next, each article was analysed to remove those not fulfilling the delimited inclusion criteria.

Studies were eligible if all the following conditions were verified: (1) they pursued any prognostic or preventive health-related objective, (2) noninvasive objective physiological assessment methods were applied, (3) physiological monitoring systems were used to continuously collect physiological data, (4) procedures were developed within an active working-age population from a specific occupational group and (5) physiological measurements were developed during real-life activities, representative of a particular occupational group. This process was carried out independently by two reviewers (D.B. and J.C.G.), and a third (J.S.B.) resolved discrepancies.

Full-text was retrieved from the final studies to collect information of interest by using a customised table. Compiled information mainly involved: reference and country, study goals, measured variables and equipment, sample characteristics (size, gender distribution, mean age range), occupational group, context and duration of experimental protocols, data processing methods and respective conclusions.

Finally, to determine the strengths and weaknesses of every study’s design and their influence on corresponding results, each article was assessed for risk of bias in two phases. First, each study’s general characteristics were analysed following the sought goals of this review and considered objectives, assessed variables, applied methods and equipment, assessment procedure and measurement time. Later, referencing the Cochrane Collaboration’s Tool [32] and additional literature sources [33,34,35,36], methodological issues were addressed: ethical standards fulfilment, sample justification, clear description of the experimental procedure and practical difficulties. For this purpose, a customised table was elaborated to examine each article according to 25 items referring to seven topics: study design, participants, data sources, reporting bias, limitations, generalisability and potential sources of additional bias. Criteria were determined to address methodological difficulties and potential risks of bias of obtained results.

The study design topic involved seven questions on the rationale and clearness of defined objectives and outcome measures, the existence of a control group, the research design, description of methods and equipment and reporting of statistical analysis. The participants category was determined by five questions and verified if ethical standards were met, if the sample size was justified and a randomisation method was performed. It examined whether subjects were similar at baseline regarding the most significant indicators and whether the number of subjects was representative to assure the study’s statistical power. Regarding the data sources, two criteria checked the inclusion of real-life working activities within the experimental protocols and assure all subjects completed all testing.

Similarly, six questions in the reporting bias section determined if sample characteristics were adequately provided and withdrawals and dropouts were registered. It also verified if the available literature supported numerical data, if complete outcome data were provided, tables and figures were clearly presented and if data supported the study conclusions. Two additional criteria were added in the limitations and generalisability categories, determining if limitations were objectively defined and if the study allowed results generalisation. Finally, potential sources of bias were determined by three questions and identified additional causes of bias related to withdrawal or dropout rate of participants, number of missing outcomes and practical difficulties.

Each item was marked with Y (yes), N (no) or U (unclear) for the cases in which there was not enough information to define whether the criteria had been met. Then, rates were calculated by averaging the number of positive answers from each category and adding up their results. To simplify the interpretation process, obtained scores (0–7) were then transformed and presented as percentage values (0–1), with studies with higher values considered the most suitable for the objectives of the current review. Conversely, investigations with a higher rate of negative answers (scores under 50%) would immediately be considered for exclusion. This process was performed by two reviewers (D.B. and J.C.G.) and was verified by a third (J.S.B.). Due to the differences in study protocols (assessed variables, recordings duration, occupational groups, experimental protocols and measurement conditions) and the lack of a comparator, a meta-analysis could not be performed, with results being tabulated and described narratively.

## 3. Results

### 3.1. Studies Selection

**Figure 1:**
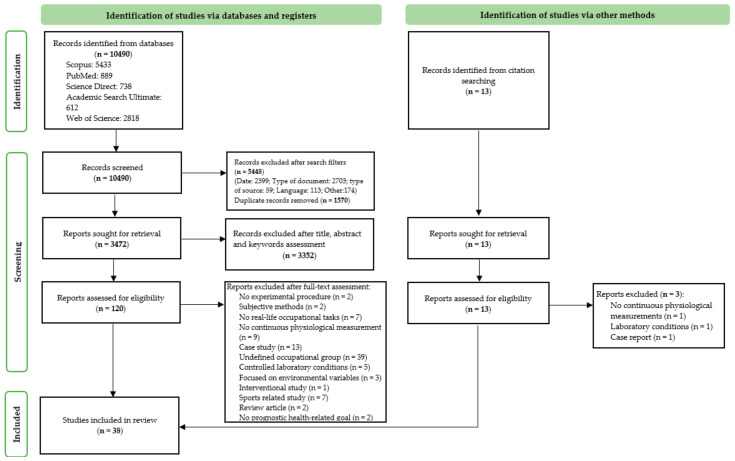
Summary of the research, based on PRISMA Statement flow diagram [38] and protocol [30].

By following the PRISMA Statement, 10,490 first items were retrieved from the databases searches and using the search engines’ filters, restrictions of date, article type, source type and language were applied. The adapted query and filters for each database are presented in Table 1. After concluding this phase, 5042 articles were identified, of which 1570 were duplicates, leaving a total of 3472 articles for a third phase. During this stage, 3352 articles were excluded for not being applied within a specific workers’ sample or involving invasive or subjective measuring methods, and 120 articles were left for a final full-text assessment. Lastly, following the eligibility criteria, 28 relevant publications were identified, with the references from these 28 studies being retrieved and additional articles found according to the inclusion criteria. This process was repeated in the newly selected items as preconised by the snowballing technique [31], and 10 more articles were gathered, leaving 38 articles for analysis in the current review. Figure 1 provides an overview of the number of studies from each PRISMA methodology stage [37].

### 3.2. Characteristics of the Included Studies

**Figure 2:**
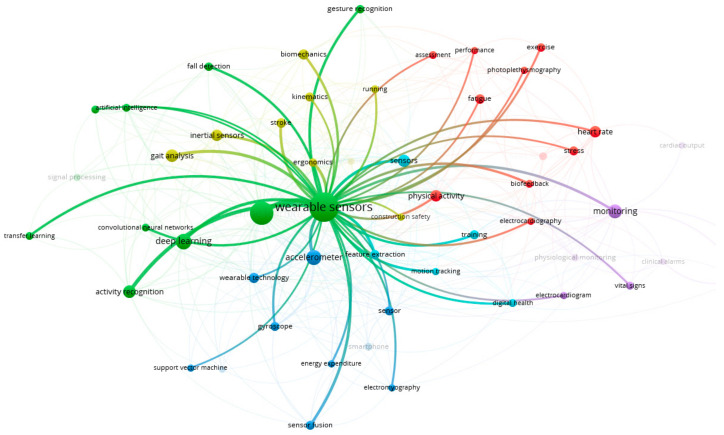
Keywords connections extracted from Scopus and VOSViewer.

**Figure 3:**
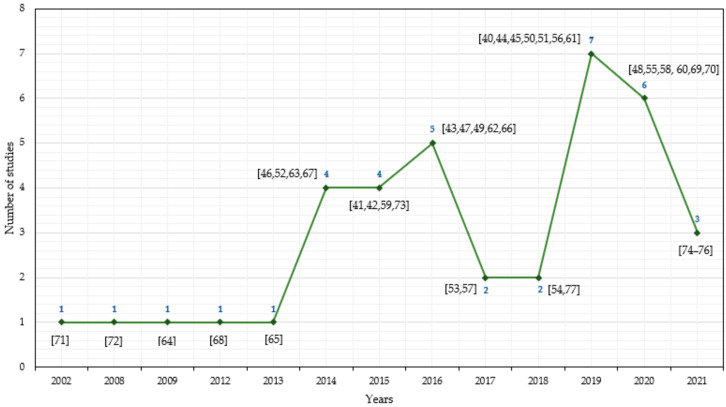
Selected articles grouped by year (until 11 October 2021).

Figure 2 shows the network visualisation of some controlled terms automatically identified across studies. With data extracted directly from Scopus after inserting the criteria outlined in Table 1, 54 relevant terms in six clusters were identified through VOSviewer [39]. These terms correspond to the authors’ keywords with at least 10 occurrences throughout all retrieved studies. Regarding the final 38 articles included [40,41,42,43,44,45,46,47,48,49,50,51,52,53,54,55,56,57,58,59,60,61,62,63,64,65,66,67,68,69,70,71,72,73,74,75,76,77], Figure 3 provides an overview of the evolution of the studies using physiological monitoring systems over the years (until 11 October 2021). Research with these systems is growing among occupational groups, and from 2019, the increasing trend is even more notorious.

**Figure 4:**
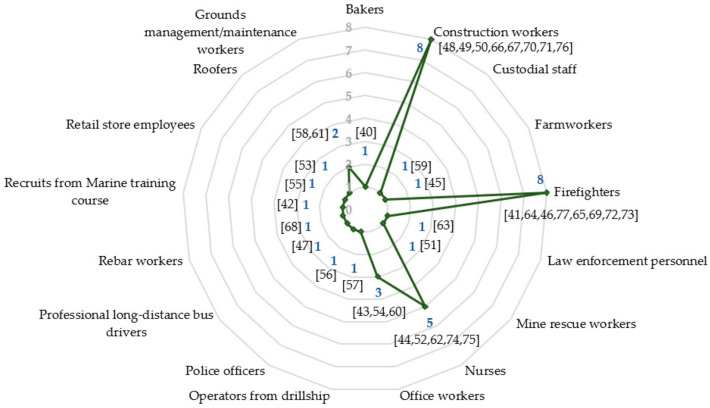
Number of included studies categorised by occupational groups.

Concerning the included professions, since there were no restrictions applied in this regard, various occupational groups (based on the minor groups level from the International Standard Classification of Occupations ISCO–08) were retrieved and, as Figure 4 illustrates, there is an evident focus on firefighters and construction workers. In terms of gender distribution, a more considerable percentage of male subjects was observed since 23 out of the 38 selected papers recruited only male workers (only one study focused on a female sample). Sample sizes were diverse, ranging from six roofers to 134 office workers. All participants were part of the healthy active working population and their mean age values ranged from 20 to 46 years old. All used comparisons with previous or basal levels of the same subjects. Most studies (*n* = 34) considered real working scenarios only, while four performed measurements combining real and controlled laboratory conditions.

**Figure 5:**
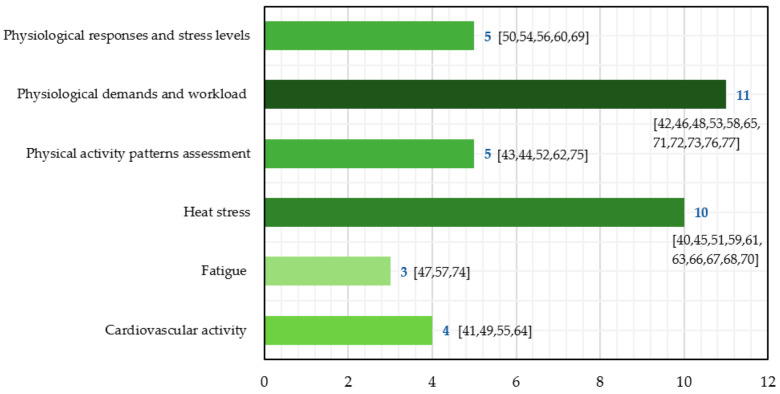
Included studies based on their health and safety-related objective.

Despite the diversity of the 38 included studies, six research goals areas could be identified, including examining the effects of heat stress exposure, measuring the physiological demands and workload associated with specific working activities, determining the stress levels experienced by workers, evaluating heart rate-derived variables, addressing fatigue levels and its methods of assessment, and monitoring working postures and activity patterns (sleep disorders and physical activities classification). As Figure 5 shows, a clear tendency of assessing physiological demands and workload, as well as heat stress from particular occupational activities and environments, was observed. In some cases, studies could be associated with more than one of these groups and were placed in the group considered closest to its objective.

Selected studies are presented in Table 2, grouped by their general research objective and covered professions. Measured physiological variables (continuously or not) and the other data collection approaches divided into biochemical, subjective, cognitive and environmental variables are also summarised. First, the aim was to identify the trends and applications when monitoring physiological variables and the addressed occupational groups. Subsequently, the sensors used to measure the physiological responses were analysed, with their list being presented in Appendix A (indicating available information on the sampling frequency, sensor’s accuracy and location).

From the extracted information, the most notable outcome is related to cardiovascular activity monitoring, with only six [43,44,47,52,62,74] from the 38 studies not including one or more heart rate derived variables in their analyses. For this purpose, the chosen equipment consisted mainly of Polar and Garmin heart rate monitors and Equivital, Zephyr and Ecgmove multivariable signals monitors. On the other hand, thermal responses (skin or core body temperature) were identified in 11 of the 38 publications [40,42,45,50,51,65,68,69,72,73,76]. Core temperature was measured with ingestible thermometric pills and skin temperature from various body parts was mainly collected using Equivital LifeMonitors and E4 Empatica wristband-type sensors.

Respiratory rate was examined in six works [45,47,51,56,68,69] through Equivital and Zephyr monitors, among others. Similarly, physical activity patterns were monitored in 14 studies [42,43,44,52,53,57,59,61,62,69,70,73,74,75] by using tri-axial accelerometers from ActiGraph, Fitbit and GeneActiv. Concerning the processing and analysis of these variables, traditional methods were the most recurrent approach. Most of the articles recurred to statistical tests to examine the data and present outcomes. Less observed was the use of customised algorithms and machine learning methods [47,50,62,76].

### 3.3. Risk of Bias Assessment and Quality of Results

**Figure 6:**
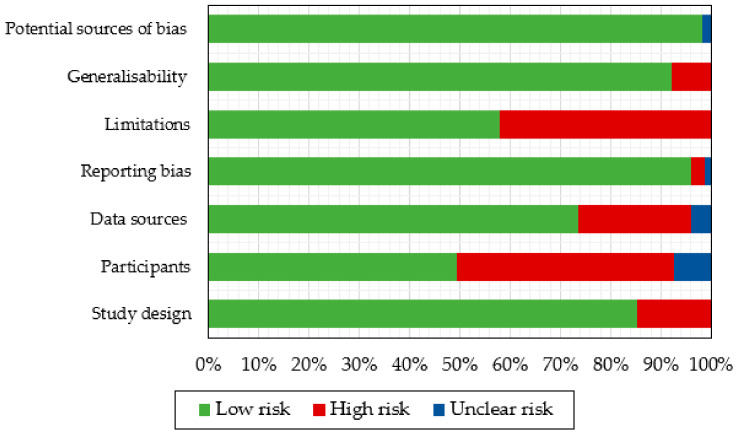
Risk of bias assessment overall results.

A customised table was used to assess the risk of bias and results quality, with each article being analysed according to 25 items from seven categories. Criteria aimed to address methodological difficulties and potential risks of bias of obtained results. Results per category are compiled in Figure 6 and detailed in Appendix B. Overall, studies were rated as positive in quality since they presented scores above 50% and, no article was considered for exclusion. Still, methodological weaknesses were observed in all of them and no article reached the highest score. Some of these weaknesses were related to the studies design, with no study including control groups, and two not providing details on the performed statistical analysis. Both factors do not necessarily mean biased results, but they would certainly enrich their quality and generalisability.

In addition, only three investigations [40,41,42] declared randomisation procedures in the participants category, and none described power adjustment or consideration to account for the adequacy of sample size. For four cases [48,53,65,76], with samples under 10 participants, the number of subjects was not considered representative to assure statistical power in the study. As part of the data sources evaluation, nearly half of the papers [42,43,44,45,46,48,49,51,52,54,56,58,60,63,72,73,77]) indicated that not all subjects homogeneously completed all parts of the protocols. Concerning any form of reporting bias, most of the criteria were fulfilled and, weaknesses were found when verifying the description of withdrawals and dropouts in six studies [40,41,44,66,67,70]. Finally, the limitations assessment revealed that 16 out of the 38 studies [40,41,42,44,47,49,50,55,57,59,63,67,68,71,72,73] did not describe the limitations and opportunities to improve their developed investigations.

## 4. Discussion

This review focused on assessing continuous physiological responses during working activities to systematise the evidence of physiological monitoring applications for occupational settings through noninvasive procedures. Studies goals were mainly to quantify the impact of specific physically demanding tasks and examine the heat stress associated with some occupational environments. The analysis from VOSviewer (Figure 2) gave a first glance at the results and studies tendencies. However, no strong connection between the terms was found, and only six clusters were formed among them. Still, the main term “wearable sensors” was related with “heart rate” (including “heart rate variability” and “cardiac output”), “physical activity” and “motion tracking”, their respective measurement methods “accelerometer”, “electrocardiogram”, “photoplethysmography” and potential examined conditions “performance”, “fatigue” and “stress”. Within the 38 articles, these terms were relevant and consistent with the investigation line and identified goals.

### 4.1. Monitored Physiological Variables

#### 4.1.1. Cardiac and Thermal Responses

Results revealed the cardiac responses to specific occupational activities as the most considered monitoring approach. Consistently, a vast amount of available literature has evidenced heart rate as the most widely used form of physiological information for personal health conditions [78,79]. Based on current occupational physiology research, this goal can be explained since this variable is sensitive to various work-related conditions such as changes in physical and mental fatigue [80,81] and sleep and circadian issues [82,83]. Furthermore, heart rate variability has been identified as a valid measurement of autonomic nervous system regulation [84] and is influenced by other physiological systems, particularly the respiratory, endocrinological and immunological [85]. Authors also stated that these variables (heart rate and heart rate variability) have both time and cost advantages over other methods, such as biomarker testing, being also noninvasive [12]. Specifically, heart rate variability has been previously concluded to be the most useful physiological metric for fatigue measurement among some occupational groups [85]. However, in this regard and despite its proven usefulness, other authors indicated that further studies are still needed to prove that this variable can improve fatigue monitoring [26,86].

Studies also monitored thermoregulatory responses and physical activity patterns based on accelerometry to improve heart rate evaluation accuracy. Authors proved the relevance of combining thermal and cardiac responses since their results together were more accurate than individually [87,88]. According to normative guidelines [89] and available literature [90,91], cardiac and thermal measurements are not conclusive indicators by separate since the interaction among environment, physical demands, personal protective equipment, anthropometrics and other individual and contextualised factors are multifaceted [92]. However, these variables combined are reliable markers of various occupational risks (e.g., acute stress, physical exertion and heat strain) and health associated effects. During intense physical activity (as in most referred professions), the human core body temperature rises and, through thermoregulatory changes, the body tries to maintain its core body temperature. Hence, by exploring the patterns of this variable, comprehension of physical demands could be advanced [88].

#### 4.1.2. Other Monitored Variables

Occupational physical activity or work-related physical activity (generally measured from tri-axial accelerometers) was found of particular and ongoing interest since it can be related to various health risks for workers if completed improperly, repetitively or in the absence of remedial measures [93]. According to retrieved results, physical activity monitoring is an effective approach in the work environment, especially when combined with physiological data collection, with the selection of the most suitable physiological parameters for monitoring depending on the specific research objective.

Alternatively, respiratory variables also provided useful information about physical exertion and fatigue during occupational activities. Among the analysed studies, they were used for measuring the physiological cost of working activities [51,67], although none utilised them for physical exertion modelling. The respiratory rate has relevant implications for different fields (from survivor identification in civil and military scenarios to examining outdoor activities’ physical effort and, even, as an indicator of emotional or cognitive load [94]), with recent studies relating it more to physical effort than heart rate and oxygen consumption under various experimental conditions that affect fatigue development (e.g., hypoxia, muscle fatigue and heat exposure) [95,96]. The use of respiratory rate information combined with heart rate variability has been previously addressed for stress detection and management [97]. Studies that monitored this variable have proven its usefulness, but further research is needed to deeply explore the reported previous outcomes within occupational environments.

In general, the included studies’ findings described how physiological monitoring could report diverse occupational health risks and how these variables respond differently based on individual differences. Monitoring physiological responses in real-time would allow for the possibility of adaptations to optimise an individual’s performance in the face of the developed task and according to his capabilities [98]. Combined with contextual data, physiological variables provide valuable tools for enhancing assessment procedures adapted to tasks’ characteristics. As literature shows, a generalised approach is insufficient to assess occupational stress on an individual level [99], meaning that wearable technology might be a good alternative since it increasingly allows obtaining continuous physiological data from individuals during training or working conditions. However, data must be combined with the subjects’ and contextual characteristics, and translated into simple and actionable information for both workers and leaders.

### 4.2. Physiological Monitoring Systems and Processing Methods

Several methods for continuous physiological monitoring have been developed, accepted among various research fields and used or feasible in the workplace to reduce the risk of work-related health impairments. Observing the selected articles, the simultaneous use of sensors for multivariable continuous measurements was a clear tendency. A variety of devices were identified, and, as Appendix A details, most of them referred to integrated physiological monitors able to capture multivariable signals simultaneously. These devices included the Equivital LifeMonitor [21] (a multivariable signal device with ECG and respiratory monitor, inbuilt medical-grade thermometer and tri-axis accelerometer), identified in seven studies [42,51,57,63,69,73,77], and the Zephyr status monitor (including ECG, skin temperature, breathing rate and tri-axis inertial signals) observed in three [45,53,56]. Wristband-type devices such as the E4 from Empatica (including PPG, electrodermal activity, skin temperature and tri-axis signals) and the Basis Fitness (PPG and tri-axial signals) were also used in two studies each [49,50,59,76].

Cardiac signals were mostly measured with different versions of the Polar heart rate monitor [100] (eight studies [40,49,65,67,68,70,71,72]), while ActiGraph [101] and Fitbit [102] accelerometers were found in three studies each ([53,62,73] and [70,74,75], respectively) to report physical activity characteristics. Regarding core temperature monitoring, ingestible thermometer pills were used in three studies [51,65,73]. Furthermore, e.g., for heat stress assessment, additional equipment such as portable weather meters were used.

Most of the physiology data were computed through posterior statistical analysis, an approach that makes its applicability for real-time prediction unfeasible. The most recent approaches included customised and supervised machine learning algorithms (such as Hidden Markov models and Gaussian support vector machine algorithms) to classify different fatigue levels, heat stress and physical activities intensity. Regarding how data are presented, some studies used personalised mobile applications to summarise outcomes and alerts from health monitoring [59] and provide real-time feedback. One study even addressed the positive health effect of periodically reporting this information to workers [58]. These examples help evidence the evolution in data management. The trend continues in assessing traditional fatigue and physiological workload indicators (heart rate, thermal responses, respiration signals). The difference lies in using the new available computational techniques, giving rise to a new generation of effective non-intrusive mechanisms to monitor health variables.

### 4.3. Safety and Health Applications

**Figure 7:**
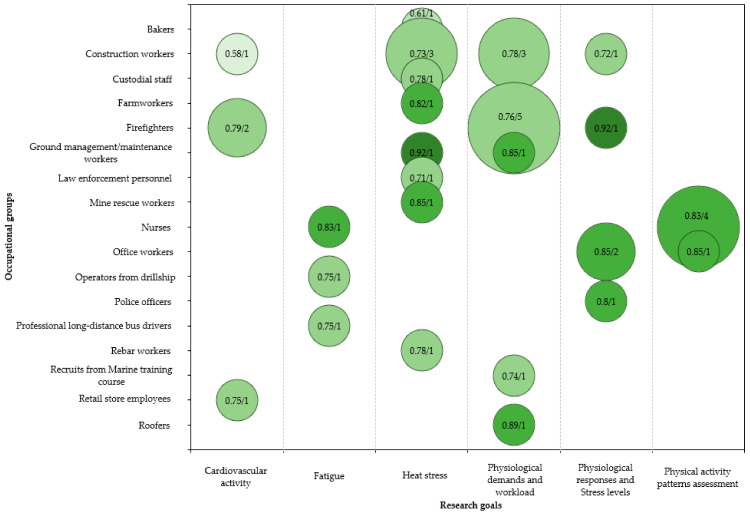
Map of reviewed articles based on research goals and occupational groups. Each bubble represents the number of studies under each category (size) and the results from the risk of bias assessment (colour scale).

Six general research areas were identified, and Figure 7 provides an overview of the number of studies related to each, categorised by the occupational groups assessed. The size of each bubble is proportional to the number of studies found within each specific objective and occupational group. The green colour scale represents the scores (0–1) obtained from the risk of bias assessment, increasing the colour intensity as it approximates to 1. The average score and number of studies are presented inside each bubble.

#### 4.3.1. Thermal Stress

Within studies one common goal was to examine the physiological responses of heat stress exposure among various professions (e.g., construction [66,67,70], mining rescue workers [51] and farmworkers [45]). Results reported that when workers develop activities outdoors (as in most examined cases), the combined application of environmental and physiological measurements is the best alternative for evaluating heat stress and strain in hot climates. These measurements are essential not only for hot climates exposure but also for extremely cold temperatures faced by these same occupational groups [103]. However, studies from this review did not address cold assessment in the field context.

Nevertheless, literature has demonstrated the growing interest in cold exposure assessment focusing on controlled-laboratory conditions evaluation [104]. Working in outdoors arctic conditions, for example, requires protection against cold and high wind speed, while the varying ambient conditions and workload create problems in the adjustment of the thermal insulation of clothing during work. Recent studies have addressed these issues [105], but there is a current gap in physiological assessments in the field because studies examining real-life conditions usually focus on environmental or subjective evaluations [106,107]. As a result, studies express the current need for occupational heat and cold exposure real-time assessment methods, being required to examine not only subjective scales or weather station data to characterise the effects of climate-related changes but also using physiologic endpoints at the individual level. Given the usefulness and limitations from subjective and objective (through physiological monitoring) assessments, an optimal evaluation approach would combine both to be able to have a deeper understanding on the impact of occupational demanding activities on the individual.

Research perspectives in occupational settings should always consider performing individually based assessments. For this purpose, the inclusion of both cardiac and thermal responses appears essential, but how to best measure them differs. Even if core body temperature provides the most accurate estimate of heat or cold stress effects, its continuous monitoring is not always feasible and, sometimes, not recommended (e.g., mining rescue work [51]). Alternatively, combining skin temperature with heart rate is the best proxy measure for field use in first responders at this time since the skin transfers heat from the body core to the atmosphere guided by complex thermoregulation and other physiological adaptation mechanisms. Complementarily, recent laboratory studies [108] have also addressed the potential of heart rate and skin temperature along with electrodermal response to assess work-related heat stress since this variable can be a useful indicator of bodily response to humidity, high temperatures and physical activity. However, further work is needed to determine this applicability during regular working conditions.

#### 4.3.2. Physiological Workload

Additionally, what was common among 11 publications [42,46,48,53,58,65,71,72,73,76,77] was the assessment of the physiological workload resulting from specific activities that was defined in several ways depending on the monitoring goal. However, all studies agreed on how continuous monitoring of physical load in workers can increase an understanding of their health conditions and performance effects [48,53]. In the referred 11 studies, target groups were mainly firefighters and variables such as maximal heart rate, physical activity-related variables, age and environmental variables (e.g., WBGT) were found as essential metrics for workload estimation. Some also included measurements of core temperature. Similar to what happens when assessing the effects of extreme temperatures, this variable gives an accurate representation of the internal body reactions to physically demanding activities but is not feasible to be continuously measured in all occupational environments.

Furthermore, within one of the most recent studies [76], variables such as electrodermal activity and skin temperature were also evidenced as highly informative signals to assess the effects of different workloads. Research on the applicability of these variables has increased, particularly among construction workers [25,87,108]. However, while promising, most of these investigations have been developed in controlled conditions, which indicates that their in-field application can be a short-term research perspective.

#### 4.3.3. Stress Detection

Within studies, stress monitoring was a clear assessment interest among diverse occupations (construction workers [50], police officers [56], firefighters [69] and office workers [54,60]), with five papers [50,54,56,60,69] presenting goals directly oriented in this regard. The impact of stress on health conditions is well recognised [109], and literature has evidenced how stress perceptions activate physiological responses [110], with reviews gathering findings on this topic also being developed [111,112,113]. Considering the low-cost and availability of good quality sensors, it was demonstrated that, in general, collecting data in a real environment and highly stressful occupations is feasible. Real-time psychophysiological monitoring can serve as an early screening for chronic stress symptoms and the basis for further contact with professional care. The outcomes from the current review suggest that a proper evaluation of chronic occupational stress would undoubtedly improve the prevention of stress-related disorders, enhance job satisfaction and quality of life of the employees, and decrease errors inflicted by the human factor [69].

For this aim, examined variables included mainly cardiac reactivity (heart rate, heart rate variability, percentage of heart rate reserve) and accelerometry data combined with perceived stress scales. Stress assessment using this multivariable psychophysiological approach was indeed concluded as a more reliable alternative for monitoring and even early screening of chronic stress than using any of the variables by separate. Consistently, literature from the current and other related reviews showed that, despite its undeniable merits, stress assessment based solely on heart rate metrics (the most common method to assess the impact of stress and various related conditions) is not accurate enough since several factors affect it, like the circadian rhythms, physical activities and body position [110]. Combining these measurements with data from other sensors could explain differences in heart rate measures among various psychophysiological states. The inclusion of accelerometric data helps control physical activity by assessing the type of motion [69].

Other identified variables included noninvasive variables: electrodermal activity [50], skin temperature [50,69] and respiratory rate [56,69]; biochemical markers such as cortisol levels [50]; and environmental conditions [60] (this last when stress may be associated with environmental factors, specifically hot or cold exposure). Finally, regarding processing methods, Jebelli et al. [50] proved that the application of supervised learning machine algorithms could be a feasible option for translating the multivariable measurements (cardiac reactivity variables, electrodermal level and response through skin temperature) into stress levels, which can contribute to assessing workers in real-time during their regular activities.

#### 4.3.4. Physical Activity Patterns

Examining activity patterns through accelerometry was observed as another important measurement goal, with five studies (four including nurses [44,52,62,75] and one with office workers [43]) focused on assessing any of these variables. Two papers monitored working postures [44,62], one examined the sedentary profiles of office workers [43], another one analysed sleep disorders while working in rotative shifts [52] and the last one addressed sleep patterns and their correlates with physical activity [75]. Actigraphs located in various body parts were the most used method. However, articles evidenced that estimates of physical activity measured solely with one or two (waist or wrist-worn) physical activity monitors may insufficiently capture physical work demands for nursing personnel and professionals with similar work characteristics. Changes in temperature, humidity and emotional stress (among other factors) may cause an increase in e.g., heart rate (observed as one reliable indicator of autonomic activity) without an increase in physical activity [62].

Furthermore, activity classification based only on counts per min may not be enough in some cases to distinguish activities with similar physical intensity. Studies have previously shown that it is possible to classify specific behaviours based on a single wearable sensor [114] and even estimate the energy requirements through various prediction methods [115]. However, more detailed sensor information is needed for accurate activity classification than one value per min, such as high-frequency data or additional sensors such as gyroscopes [14]. These more complex sensing and analysis methods often need calibration for groups or individual subjects, which hinders the comparability of reported behaviour patterns [43]. Therefore, activity monitors provide valuable information to understand rhythms and predict active and sedentary behaviours. However, they require additional physiological or contextual variables to make the outcome useful in innovative physical activity interventions towards healthy behaviours.

#### 4.3.5. Cardiac Activity

Most articles used at least one heart rate derived variable in their assessments and four had goals directed mainly to examine their responses. Heart rate is an essential indicator of physical activity and overload [116,117], with its rate variability being demonstrated as an effective health and performance tool in tactical environments [12,118]. Electrocardiography and photoplethysmography were the applied methods, and, various metrics from both signals (heart rate, heart rate variability and percentage of heart rate reserve) were extracted to provide information on subjects’ physical and mental health. Several studies proved that continuous heart rate monitoring can be used to detect any abnormal value that could result from a worker’s health problems such as fatigue, cardiovascular disease (e.g., heart valve problems, arrhythmia, heart attack and stroke), or heat-related injuries (e.g., heat stroke and heat exhaustion) [49].

In addition, it was demonstrated the feasibility of obtaining high-resolution electrocardiogram signals during physically intense activities such as firefighting [64]. The continuous field monitoring strategy of these groups could provide new insight into the association between their particular professional lifestyle and high cardiac risks. Given the characteristics of their duties, future research trends should develop algorithmic guidelines designed to route at-risk professionals for optimal cardiac care to reduce their modifiable cardiovascular risk factors.

#### 4.3.6. Fatigue

Human fatigue reduces physical activities’ capability because of preceding physical exertion or excessive physical overload [25]. It degrades performance and health, causing errors, incidents and accidents in operational contexts, and has been extensively studied through both subjective and objective methods [1,26,118]. However, despite its prevalence and well-studied consequences, a fatigue objective physical manifestation has not been well documented in occupational settings [119]. Occupational fatigue research has almost exclusively implemented subjective assessments in questionnaires, primarily based on self-reports dealing with perceived fatigue and outcomes based upon work-related incidents. Such subjectivity is not representative of actual human performance-based functionality and can easily be manipulated to reflect the desired outcome [120]. As Mehta et al. [57] indicate, some of these standardised fatigue surveys are not always appropriate for some groups (e.g., drillship operators, analysed in their study), nor are they validated against physiological fatigue outcomes from those workers.

Among results, three articles explicitly addressed fatigue detection through physiological measurements [47,57,74] and compared responses with self-reported fatigue states. Fu et al. [47] proposed a fatigue detection model based on a dynamic Hidden Markov Model using cardiac, respiration and EMG signals simultaneously recorded from sensors and sent to a computer by Bluetooth during real driving. This approach, involving 12 professionals and 3.5 h of driving, was concluded as an effective way to make rational inferences on drivers fatigue. An equivalent physiological fusion alternative for fatigue detection was proposed by Aryal et al. [87], in a simulated environment and using boosted tree classifiers. In both cases, the combination of features from the different sensors led to a higher accuracy than using features from only one of them. These examples suggest that by integrating modern computing techniques, solid research perspectives are anticipated as these prediction models can be adapted to several occupational groups and lead the path to develop warning systems against high levels of physical fatigue and improving work-rest schedules. Nevertheless, despite its promising perspectives, the success factors of artificial intelligence implementation in real-life occupational settings have not yet been deeply evaluated, suggesting another short-term research need.

### 4.4. Current Trends and Future Research Perspectives

The different studies addressed in the current review helped to evidence the trends in research regarding the most monitored physiological variables and the usefulness of their information. While investigation using physiological monitoring systems is quickly evolving, limitations for their application and interpretation among occupational groups emerge. Some studies highlighted the need for standards suitable for application within physically demanding occupations concerning physiology data interpretation. For example, in Davis et al. [46] study, results from specific firefighting activities monitoring were compared with the American College of Sports Medicine guidelines, indicating limits of age-predicted percentage of maximal heart rate and heart rate reserve (85% and 70%, respectively) after which exercise should be stopped [121]. Both criteria were reached in most of the evaluated firefighters and evidenced the tremendous physiological cost of their activities and the need for specific guidelines or assessment methods for physically demanding occupations such as those from tactical personnel [117].

Consistently, previous studies concluded that normative guidelines (e.g., ISO 8996 [122]) were not ideal for firefighters, and two new classes for the classification of metabolic and respiratory responses to intensive work were proposed in the study of Holmér et al. [123]. Furthermore, regarding the feasibility of some measurement approaches, variables with recognised validity for heat stress assessment (such as core temperature) were of restricted applicability in some occupational settings, opening the pave for the application of alternative measurements (e.g., skin temperature) or estimation procedures such as those addressed by Falcone et al. [124].

As expected, most investigations analysed results from multivariable signals through statistical analysis after all data were collected and evidenced that the real-time analysis of sensory data should be undoubtedly the focus of future research. On the other hand, limited evidence was also found in applying other processing methods such as machine learning techniques. Supervised learning algorithms including dynamic Markov models and Gaussian support vector machine were used with physiological, motion and contextual data to allow an efficient classification of events (e.g., stress and fatigue levels). The advantage of these and other similar processing methods is that they can continuously process large data sets and provide an easy understanding of physiological information, allowing individualised assessments and timely interventions in the workplace. In addition, the application of these models also proved that the simultaneous analysis of multiple physiological signals can improve the accuracy of their predictions and reduce their sensitivity to errors. Research perspectives should deeply explore the applicability of these machine learning algorithms conducting real-time assessments while combining the data from multiple wearable sensors to accurately describe workers’ well-being and health conditions.

As findings revealed, it is fundamental to understand the causes behind workers’ unsafe behaviours to remove these root causes that do not help manage their job demands. By leveraging wearable sensors and regularly obtaining data at the individual level, it is possible to eventually explain how effectively and positively a worker’s physiological reactions can change his/her job demands, as well as safety and productivity performances [53]. In summary, the tendency to use validated variables and procedures for physiological measurement is maintained for occupational applications and, future perspectives should be oriented to validate the other referred variables and processing methods within more extensive samples and during real-life operations.

### 4.5. Limitations

Despite the obtained results, limitations at the review level include language bias since studies in languages other than English were not considered, as well as publication bias because no unpublished research was included. The potential exclusion of some articles due to the applied rigorous criteria could have left out data sources and, the experimental procedures heterogeneity (in working tasks, assessed variables, used approaches and data processing methods) did not allow conducting a statistical analysis combining the results. In addition, criteria for assessing the methodological quality and bias may not have been the most suitable for all articles. Given the high variability across studies, only the most notable research goal was considered when classifying health-related objectives. Even though rigorous classification schematised and structured the obtained results providing a clear understanding of up-to-date research orientations, identified research objectives may have disregarded other relevant specific study goals of some papers.

## 5. Conclusions

The current review reports on recent investigations of continuous physiological monitoring for quantitative assessments with occupational health-related goals. Responding to each of the addressed research goals from this study, the following conclusions could be gathered:

(i) Cardiac variables (specifically heart rate and heart rate variability) were identified as the most used physiological metrics, providing useful information about various domains affecting workers performance and wellbeing. However, findings also evidenced the need to use these variables with other metrics and contextualised individual information to diagnose the studied condition. As a result, skin and core temperature were proved essential for thermal stress and fatigue assessment and physical activity based on accelerometry for determining activities workload. Furthermore, the limited (but promising) evidence on respiratory-related variables associations and electrodermal activity were pointed as the potential focus in future research.

(ii) Among the included studies, six research objectives were identified and helped delineate the overall perspective on the research trends among occupational groups. Heat stress assessment and the quantification of physical demands and workload of specific activities were the most recurrent. Additional studies were found focusing on the cardiovascular responses to intense and stressful working activities. Fatigue and stress assessments were also addressed, while various conditions associated with physical activity patterns were evidenced mainly among nurses. The information extracted and analysed from each study helped observe which physiological variables were used for each area and occupational group.

(iii) The latest approaches and systems for measuring human physiology among occupational groups were retrieved and highlighted using multivariable signals sensors. The evolution and applicability of these systems were corroborated, but the evidence on real-time processing approaches was found limited.

(iv) Challenges for future research are centred on the processing methodologies of physiology information and the application of principled computational techniques that allow continuous and real-time monitoring in operational settings, promoting to sustain workers’ given tasks more safely and healthily.

As a result, this review established various directions for further research. It can be the starting point for future in-field investigations by providing a general view on the up-to-date related research among occupational groups. Furthermore, it can derive into more in-depth reviews addressing any of the six different research domains in both laboratory and field conditions. Finally, the limited evidence on the applicability of some physiological metrics and real-time processing methods can be the focus of further investigations.

## Figures and Tables

**Table 1:** Summary of articles after databases filtering.

Database	Adapted Query and Database Filters
Scopus	((TITLE-ABS-KEY(“physiolog* monitor*”) OR TITLE-ABS-KEY(“noninvasive monitor*”) OR TITLE-ABS-KEY(“medical monitor*”) OR TITLE-ABS-KEY (“wearable sens*”)) AND (TITLE(assessment) OR TITLE-ABS-KEY(occupational) OR TITLE(model) OR TITLE-ABS-KEY(fatigue) OR TITLE(algorithm) OR TITLE-ABS-KEY(worker) OR TITLE-ABS-KEY(training) OR TITLE-ABS-KEY(“physical exertion”))) 2014–2022/Article, Article in Press/Journals/English
PubMed	((“physiological monitoring”[All Fields]) OR (“noninvasive monitoring”[All Fields]) OR (“wearable sensor”[All Fields]) OR (“medical monitoring”[All Fields])) AND ((assessment[Title]) OR (occupational[All Fields]) OR (model[Title]) OR (“fatigue”[All Fields]) OR (algorithm[Title]) OR (worker[All Fields]) OR (“training”[All Fields]) OR (“training”[All Fields]) OR (“physical exertion”[All Fields])) 2014–2021/Journal Article/English/Humans
Science Direct	(“physiological monitoring” OR “noninvasive monitoring” OR “wearable sensors”) AND (TITLE(assessment) OR occupational OR TITLE(model) OR fatigue OR TITLE(algorithm) OR worker) 2014–2022/Research articles/Subscribed journals
Web of Science	(TS = (“physiolog* monitor*”) OR TS = (“noninvasive monitor*”) OR TS = (“wearable sens*”) OR TS = (“medical monitor*”)) AND (TI = (assessment) OR TS = (occupational) OR TI = (model) OR TS = (fatigue) OR TI = (algorithm) OR TS = (worker) OR TS = (training) OR TS = (“physical exertion”)) 2014–2022/Article/English
Academic Search Complete	(AB “physiolog* monitor*” OR AB “noninvasive monitor*” OR AB “wearable sens*” OR AB “medical monitor*”) AND (TI assessment OR AB occupational OR TI model OR AB fatigue OR TI algorithm OR AB worker OR AB training OR AB “physical exertion”) 2014–2021/Academic journals/English

**Table 2:** Studies characteristics grouped by their general research objective and covered professions.

Research Objective	Occupational Groups	Physiological Variables		Secondary Variables			References
		Continuously Measured (Main Variables)	Uncontinuously Measured	Biochemical	Subjective and Cognitive	Environmental	
Cardiovascular activity	Construction workers	HR	N/A	N/A	N/A	N/A	[49]
	Firefighters	HR, HRV, ECG	N/A	N/A	N/A	N/A	[41,64]
	Retail store employees	HR, HRV	N/A	N/A	N/A	N/A	[55]
Fatigue	Operators from drillship	HR, accelerometer counts	N/A	N/A	Fatigue subjective scales	N/A	[57]
	Professional long-distance bus drivers	EEG, EMG, respiration signals	N/A	N/A	Self-reported fatigue states	N/A	[47]
	Nurses	From accelerometry counts: sleep duration, number of awakenings and sleep latency at night; number and distribution of steps taken during the work shift	N/A	N/A	Fatigue levels using the Brief Fatigue Inventory	N/A	[74]
Heat stress	Bakers	HR	Tympanic body temperature	N/A	N/A	Natural wet temperature Tnw and globe temperature Tg	[40]
	Construction workers	HR, energy expenditure, oxygen consumption, physical work activity, fluid intake	Resting blood pressure	Pre- and post-shift urine specific gravity (USG)	RPE	Dry-bulb temperature, wet bulb temperature, globe temperature, Indoor and outdoor heat exposures (WBGT)	[66,67,70]
	Custodial staff	HR, physical activity patterns from accelerometer counts	N/A	N/A	N/A	Ambient temperature and humidity	[59]
	Farmworkers	BR, HR, skin temperature, core body temperature (estimated from skin temperature), kilocalories burned per hour	Baseline blood pressure	Serum glucose and serum osmolarity	Heat-related illness symptoms	WBGT	[45]
	Grounds management workers	HR, activity patterns	N/A	N/A	N/A	Individually experienced temperature	[61]
	Law enforcement personnel	HR, core temperature (estimated), physiological strain index	N/A	N/A	Self-reported thermal discomfort	N/A	[63]
	Mine rescue workers	HR, BR, energy expenditure, oxygen consumption, core temperature and skin temperature	N/A	N/A	N/A	Mining environmental conditions	[51]
	Rebar workers	HR, energy expenditure, BR, METs, minute ventilation, oxygen consumption, and respiratory exchange ratio	Ear temperature	N/A	RPE	N/A	[68]
Physiological demands and workload	Construction workers	ECG (smart clothing), HR, electrodermal activity, photoplethysmogram (PPG), skin temperature, 3-axis acceleration, oxygen consumption	N/A	N/A	N/A	Air temperature, relative humidity, WBGT	[48,71,76]
	Firefighters	HR, air consumption, core temperature, activity-based accelerometry counts, maximum oxygen uptake, ECG, speed and elevation gain	N/A	Complete blood count and differential cell count; electrolyte, muscle and liver enzymes; blood glucose, creatinine, partial thromboplastin and urine osmolarity	Body part discomfort, self-perceived conditions, RPE, perception of respiratory distress, thermal Sensation Scale, overall wellbeing (feeling scale)	N/A	[46,65,72,73,77]
	Grounds maintenance crew workers	HR	N/A	N/A	N/A	Ambient temperature, ultraviolet exposure	[58]
	Recruits from Marine training course	HR, activity-based accelerometry counts, and skin temperature measurements	N/A	N/A	Vigilance and memory evaluations	N/A	[42]
	Roofers	HR, HRV, activity through accelerometry data, energy expenditure, metabolic equivalents (METs), sleep quality	N/A	N/A	Self-reported productivity loss	N/A	[53]
Physiological responses and Stress levels	Construction workers	cardiac reactivity (HR, IBI, HRV, HRR), electrodermal level and response (from skin temperature)	N/A	Cortisol levels in saliva	N/A	N/A	[50]
	Firefighters	HR, activity based on tri-axial acceleration counts, skin temperature, BR	N/A	N/A	Self-assessed stress	N/A	[69]
	Office workers	Cardiac reactivity, physical activity, sleep quality	N/A	N/A	Perceived stress	Relative humidity	[54,60]
	Police officers	HR, HRV, BR	N/A	N/A	Self-reported stress	N/A	[56]
Physical activity patterns assessment	Nurses	Activity, posture and sleep patterns from acceleration counts, circadian rhythm parameters and sleep quantity; angular displacement waveforms of upper arm elevation and trunk flexion/extension; HR	N/A	N/A	Emotional and physical wellbeing; behavioural variables (sleep quality, affect, anxiety, life satisfaction, personality)	N/A	[44,52,62,75]
	Office workers	Activity based on accelerometer counts	N/A	N/A	N/A	N/A	[43]

Heart rate (HR), electrocardiogram signals (ECG), heart rate variability (HRV), photoplethysmography (PPG), oxygen uptake (VO_2_), ratings of perceived exertion (RPE), electroencephalogram signals (EEG), electromyogram (EMG) and breathing rate (BR).

## Data Availability

Not applicable.

## Appendix A

**Table A1:** Physiological sensors used for continuous monitoring among included studies.

Signals Acquired	Physiological Monitoring Systems	System Specifications/Key Features	Reported Measurement Characteristics	Number of Studies	References
Cardiac only	Polar heart rate monitors (Polar Electro, Kemple, Finland): Team Pro, H7, S625X, RCX3, Vantage XL, S710	- Water-resistant - Operating temperature −10 °C to +50 °C - Compatible with other sensors	Not reported	8	[40,49,65,67,68,70,71,72]
	H12+ digital Holter recorder V3.12 (Mortara Instrument, Milwaukee, Brookfield, WI, USA)	- 64 mm × 98 mm × 25 mm; - 12-lead ECG Pacemaker detection with ECG display and lead quality check	Not reported	3	[41,64,72]
	Garmin Smartwatch (Garmin International, Olathe, KS): Forerunner 110 monitor and Vivoactive HR	- Waterproof - Fitness tracking - Rectangular flat dial design	Not reported	3	[46,58,61]
	Apple Watch Series 1	- Aluminium case with Sport Band 38 mm × 42 mm - Splash resistant - Battery up to 18 h	Not reported	1	[55]
	Heart rate sensor myBeat WHS-2 (Union Tool Co., Ltd., Shanghai, China)	- Resolution of 1 kHz - Interval per beat - Analysis of R-R interval	5-min interval	1	[48]
Respiratory only	Open-circuit spirometry system (K4b2, Cosmed Srl, Rome, Italy)	- Gold standard wearable metabolic system - Sampling period: breath by breath - Battery autonomy: 3 h	Not reported	4	[66,67,68,73]
	AeroSport KB1-C ambulatory metabolic analysis, open-circuit spirometry-based system (AeroSport, Inc., Ann Arbor, MI, USA)	- Contains electronic instrumentation, battery, oxygen and carbon dioxide sensors, and telemetry connections to a microprocessor that permits radio transmissions of up to 300 m to a receiver and computer, can be programmed at 20, 40, or 60-s intervals - Compact (7.5 cm × 15 cm × 5 cm) and lightweight (1.13 kg)	20-s measurement interval	1	[71]
Multivariable signals	Integrated monitor Equivital Life Monitor EQ-01 and EQ-02 (Hidalgo, Cambridge, UK)	- Lightweight and optimised for long-wear comfort - Up to 48 h battery life - 9 belt sizes - Flexible software platforms	Not reported	7	[42,51,57,63,69,73,77]
	Chest-strap Zephyr status monitor	- One size shoulder strap and three strap sizes, weight: 0.64 ounces - accuracy (bpm): ±3	Not reported	4	[45,53,56,59]
	Biofeedback 2000 x-pert system	- Modular system with wireless connection	EEG, EMG and respiration modules used, 13 data sections with 15 min interval	1	[47]
	Wristband-type biosensor E4 manufactured by Empatica, Cambridge, Massachusetts	- Includes three sensors: PPG (photoplethysmography), EDA (electrodermal activity) and ST (peripheral skin temperature) - size: case 44 mm × 40 mm × 16 mm and wrist: 110–190 mm - weight: 25 g - streaming mode: 24+ h, recording mode: 32+ h, charging time: < 2 h	PPG: sampling rate of 64 Hz and an output resolution of 09 nW/digit. EDA: 4-Hz sampling frequency, 900-pS resolution, and range of 0.01–10 μS. Infrared thermopile: sampling rate of 4 Hz and accuracy of 0.02 °C within normal skin temperature	2	[50,76]
	Chest-worn sensor EcgMove 3 (Movisens)	- Combination of ECG and physical activity sensor in one compact system - Comfortable chest strap with good signal quality for long-term measurements (up to 2 weeks) - Validated energy expenditure calculation and activity recognition	Not reported	2	[54,60]
	Smart clothing COCOMI (TOYOBO Co., Ltd., Osaka city, Japan) (heart rate, respiration, perspiration, body surface temperature and joint angle)	- Underwear-type shirt integrated with biometric information sensor (detection of heart rate) and a 3-axis acceleration sensor	Not reported	1	[48]
	Samsung Galaxy S4 (Android 4.2.2 Jelly Bean Operation System, octa-core chipset, 1.6-GHz Quad + 1.2 GHz Quad CPU)	- Smartphone containing an accelerometer, STMicroelectronics LSM 330, and a Sensirion SHTC1 humidity and temperature sensor		1	[59]
	Basis Fitness Wristband: B1 and Basis Peak™ (BASIS, an Intel Company, San Francisco, CA, USA)	- Photoplethysmography (PPG) sensor for heart rate monitoring, accelerometer, thermometers (body temperature and outside temperature) and galvanic skin response (GSR) sensor	1-min interval	2	[49,59]
Activity sensor only	Promove 3D activity sensor (Inertia Technology, Enschede, The Netherlands)	- Samples and communicates wireless motion and orientation information from three-axial, fully-digital sensors: 3-D acceleration, 3-D turn rate (gyroscope) and 3-D magnetic field intensity (compass) - Data is transmitted using the low-power 2.4 GHz wireless radio to a central node, which connects to a computer through a USB - Data can also be stored in the on-board flash memory and retrieved later over USB or wirelessly	Samples of accelerations in three dimensions at 40 Hz and average sum of the Integral of the Modulus of Accelerations (IMA) per minute	1	[43]
	LSM9DS0 sensors by STMicroelectronics, a wearable microcontroller, the Adafruit Flora, via I2C	- 3D accelerometer, 3D gyroscope, 3D magnetometer - Available in a plastic land grid array package and guaranteed to operate over an extended temperature range from −40 °C to +85 °C	Not reported	1	[44]
	Wrist actigraph (Ambulatory Monitoring, Inc., Ardsley, NY, USA)	- Basic sleep estimation, high-resolution analogue data collection, simultaneous environmental data collection, subjective wearer input features, and comprehensive sleep scoring and sleep distribution data on the wrist - Lithium battery powered for long use and come with a full one-year warranty	1-min epochs	1	[52]
	ActiGraph GT9X and wGT3X-BT (ActiGraph, LLC, Pensacola, FL, USA)	- Sample rate 30–100 Hz - Battery life 14–25 days - Water-resistance 1 m, 30 min - Wear location: wrist, waist, ankle, thigh	100 Hz	3	[53,62,73]
	Accelerometer-based wrist-worn activity tracker Fitbit Flex and Fitbit Charge 2 (Fitbit Inc., San Francisco, CA, USA)	- 3-axis accelerometer and vibration motor - Operating temperature: −10 ° to 45 °C - Battery life up to 5 days, battery type: lithium-polymer	Not reported	3	[70,74,75]
	GeneActiv accelerometer wristband	- Lightweight waterproof wristband - Outputs acceleration, light and temperature data at up to 100 Hz - High resolution, raw SI unit data in an open format - Battery life: 1 week to 1 month - Body locations: wrist, upper arm, chest, waist, thigh, ankle - Operating temperature 5–40 °C - FDA 510(k) exempt, European Class 1 medical device	Not reported	1	[59]
	Philips Actiwatch Spectrum Pro	- Battery life: 55 days with four scores per day - Size: 37 × 35 × 12 mm and weight: 31 g (with band) - Waterproof (1 m for 30 min) - Operating temperature 5 to 40 °C - Contains seven data channels: white photopic light illuminance, red, green and blue light irradiance, activity, off-wrist and event marking	Not reported	1	[74]
Core temperature only	Ingestible thermometric pill (Jonah VitalSense, Respironics, Bend, OR, USA)	- Accuracy: ± 0.1 °C (32 °C to 42 °C) and ± 0.25 °C (42 °C to 50 °C) - Data resolution ± 0.01 °C - Sensing range 25 °C to 50 °C, reception range within one meter - Battery life up to 240 h active transmission - Dimensions: 23 mm × 8.6 mm and weight 1.6 g	Not reported	3	[51,65,73]

## Appendix B

**Table A2:** Methodological and risk of bias analysis of selected studies. Final scores (0–1) are presented using a green colour scale (more intense for higher scores).

Study Reference	Risk of Bias Assessment Criteria							Score:
	Study Design	Participants	Data Sources	Reporting Bias	Limitations	Generalisability	Potential Sources of Bias	
[40]	0.86	0.60	1.00	0.83	0.00	0.00	1.00	0.61
[41]	0.86	0.80	1.00	0.83	0.00	1.00	1.00	0.78
[42]	0.86	0.80	0.50	1.00	0.00	1.00	1.00	0.74
[43]	0.86	0.60	0.50	1.00	1.00	1.00	1.00	0.85
[44]	0.86	0.40	0.50	0.83	0.00	1.00	1.00	0.66
[45]	0.86	0.40	0.50	1.00	1.00	1.00	1.00	0.82
[46]	0.86	0.40	0.50	1.00	1.00	1.00	1.00	0.82
[47]	0.86	0.40	1.00	1.00	0.00	1.00	1.00	0.75
[48]	0.86	0.40	0.50	1.00	1.00	1.00	1.00	0.82
[49]	0.86	0.40	0.50	1.00	0.00	1.00	0.33	0.58
[50]	0.86	0.20	1.00	1.00	0.00	1.00	1.00	0.72
[51]	0.86	0.60	0.50	1.00	1.00	1.00	1.00	0.85
[52]	0.86	0.60	0.50	1.00	1.00	1.00	1.00	0.85
[53]	0.86	0.40	1.00	1.00	1.00	1.00	1.00	0.89
[54]	0.86	0.60	0.50	1.00	1.00	1.00	1.00	0.85
[55]	0.86	0.40	1.00	1.00	0.00	1.00	1.00	0.75
[56]	0.86	0.40	0.50	0.83	1.00	1.00	1.00	0.80
[57]	0.86	0.40	1.00	1.00	0.00	1.00	1.00	0.75
[58]	0.86	0.60	0.50	1.00	1.00	1.00	1.00	0.85
[59]	0.86	0.60	1.00	1.00	0.00	1.00	1.00	0.78
[60]	0.86	0.60	0.50	1.00	1.00	1.00	1.00	0.85
[61]	0.86	0.60	1.00	1.00	1.00	1.00	1.00	0.92
[62]	0.86	0.60	1.00	1.00	1.00	1.00	1.00	0.92
[77]	0.86	0.40	0.50	1.00	1.00	1.00	1.00	0.82
[63]	0.86	0.60	0.50	1.00	0.00	1.00	1.00	0.71
[64]	0.86	0.40	0.50	0.83	1.00	1.00	1.00	0.80
[65]	0.86	0.20	1.00	1.00	1.00	0.00	1.00	0.72
[66]	0.86	0.20	0.50	0.67	1.00	1.00	1.00	0.75
[67]	0.86	0.60	0.50	0.83	0.00	0.00	1.00	0.54
[68]	0.86	0.60	1.00	1.00	0.00	1.00	1.00	0.78
[69]	0.86	0.60	1.00	1.00	1.00	1.00	1.00	0.92
[70]	0.86	0.60	1.00	0.83	1.00	1.00	1.00	0.90
[71]	0.71	0.40	1.00	1.00	0.00	1.00	1.00	0.73
[72]	0.86	0.60	0.50	1.00	0.00	1.00	1.00	0.71
[73]	0.86	0.60	0.50	1.00	0.00	1.00	1.00	0.71
[74]	0.86	0.40	1.00	1.00	1.00	1.00	0.57	0.83
[75]	0.86	0.60	1.00	1.00	1.00	1.00	0.71	0.88
[76]	0.86	0.20	1.00	1.00	1.00	1.00	0.43	0.78
